# Remarkable variation of ribosomal DNA organization and copy number in gnetophytes, a distinct lineage of gymnosperms

**DOI:** 10.1093/aob/mcy172

**Published:** 2018-09-27

**Authors:** Wencai Wang, Tao Wan, Hannes Becher, Alena Kuderova, Ilia J Leitch, Sònia Garcia, Andrew R Leitch, Aleš Kovařík

**Affiliations:** 1 School of Biological and Chemical Sciences, Queen Mary University of London, London, UK; 2 Key Laboratory of Southern Subtropical Plant Diversity, Fairy Lake Botanical Garden, Shenzhen and Chinese Academy of Sciences, Shenzen, PR China; 3 Sino-Africa Joint Research Center, Chinese Academy of Science, Wuhan, PR China; 4 Institute of Biophysics, Academy of Sciences of the Czech Republic, Brno, Czech Republic; 5 Jodrell Laboratory, Royal Botanic Gardens, Kew, Richmond, UK; 6 Institut Botànic de Barcelona (IBB-CSIC-ICUB), Passeig del Migdia s/n, Parc de Montjuïc, Barcelona, Catalonia, Spain

**Keywords:** Gnetophytes, rDNA organization, chromosome evolution, high-throughput sequencing, concerted evolution, pseudogenes, intragenomic diversity

## Abstract

**Introduction:**

Gnetophytes, comprising the genera *Ephedra*, *Gnetum* and *Welwitschia*, are an understudied, enigmatic lineage of gymnosperms with a controversial phylogenetic relationship to other seed plants. Here we examined the organization of ribosomal DNA (rDNA) across representative species.

**Methods:**

We applied high-throughput sequencing approaches to isolate and reconstruct rDNA units and to determine their intragenomic homogeneity. In addition, fluorescent *in situ* hybridization and Southern blot hybridization techniques were used to reveal the chromosome and genomic organization of rDNA.

**Key results:**

The 5S and 35S rRNA genes were separate (S-type) in *Gnetum montanum*, *Gnetum gnemon* and *Welwitschia mirabilis* and linked (L-type) in *Ephedra altissima.* There was considerable variability in 5S rDNA abundance, ranging from as few as ~4000 (*W. mirabilis*) to >100 000 (*G. montanum*) copies. A similar large variation was also observed in 5S rDNA locus numbers (two to 16 sites per diploid cell). 5S rRNA pseudogenes were interspersed between functional genes forming a single unit in *E. altissima* and *G. montanum*. Their copy number was comparable or even higher than that of functional 5S rRNA genes. In *E. altissima* internal transcribed spacers of 35S rDNA were long and intrinsically repetitive while in *G. montanum* and *W. mirabilis* they were short without the subrepeats.

**Conclusions:**

Gnetophytes are distinct from other gymnosperms and angiosperms as they display surprisingly large variability in rDNA organization and rDNA copy and locus numbers between genera, with no relationship between copy numbers and genome sizes apparent. Concerted evolution of 5S rDNA units seems to have led to the amplification of 5S pseudogenes in both *G. montanum* and *E. altissima*. Evolutionary patterns of rDNA show both gymnosperm and angiosperm features underlining the diversity of the group.

## INTRODUCTION

Gnetophytes (division Gnetophyta) are one of the four extant gymnosperm lineages, comprising ~90 species (Doyle, 1996). Their phylogenetic placement with regard to other gymnosperms and angiosperms is controversial (Wang and Ran, 2014; Li *et al.*, 2017). Morphologically, gnetophytes share some apparent similarities with the angiosperms (e.g. vessel-like and flower-like structures, partially enclosed ovules and reticulate leaf venation), and these have given rise to the anthophyte hypothesis, which considers the gnetophytes to be sister to angiosperms (Doyle and Donoghue, 1986). However, these characters are now considered to have evolved by convergent evolution (Carlquist, 1996) and such a relationship is not usually seen in gene-based phylogenies. Instead, the most frequently recovered placement based on genomic data is that gnetophytes are either sister to or nested within the conifers (Winter *et al.*, 1999; Wickett *et al.*, 2014; Li *et al.*, 2017).

The gnetophytes themselves comprise three distinct genera, each belonging to its own family – *Gnetum* L. in Gnetaceae Lindley; *Welwitschia* Hook.f. in Welwitschiaceae; and *Ephedra* L. in Ephedraceae Dumort. (Ickert-Bond and Renner, 2016) – which are estimated to have diverged ~146 million years ago (MYA) (Lu *et al.*, 2014). These three genera are distinct both morphologically and geographically (Wang and Ran, 2014) with rather few shared characters (Ickert-Bond and Renner, 2016). The ~38 species of *Gnetum* are evergreen trees, shrubs or lianas distributed in tropical and subtropical areas across Africa, South America and South-East Asia (Won and Renner, 2005; Ickert-Bond and Renner, 2016). In contrast, *Welwitschia mirabilis* Hook f. is the sole extant representative of its family. It is a dwarf shrub that has just two foliage leaves and is endemic to the Namib Desert of south-western Africa (Namibia and Angola) (Pearson, 1908). The ~54 species of *Ephedra* are dwarf shrubs, vines, or small trees widely distributed in temperate arid areas of America and Eurasia (Price, 1996; Huang *et al.*, 2005).

This paper compares the large-scale organization and sequence diversity of ribosomal DNA (rDNA) in the three gnetophyte genera. Ribosomal DNAs contain four highly amplified ribosomal RNA (rRNA) genes, typically occurring in hundreds to tens of thousands of copies (Long and Dawid, 1980) and usually clustered at one or a few chromosomal loci. The 35S rDNA (called 45S rDNA in animals) typically contains three tightly linked rRNA genes (18S–5.8S–26S). These genes are separated by internal transcribed spacers (ITS1 and ITS2) and intergenic spacers (IGSs). The fourth gene is called 5S rRNA, and it usually forms separate arrays at chromosomal loci that are independent of the 35S rDNA in plant genomes. This is called the S-type arrangement of rDNA and, when organized in this way, the individual 5S rRNA genes are separated by a non-transcribed spacers (NTS) of variable length (Hemleben and Grierson, 1978; Ellis *et al.*, 1988; Campell *et al.*, 1992; Gorman *et al.*, 1992; Wicke *et al.*, 2011). Less commonly, the 35S and 5S rDNA sequences are linked to form 35–5S rDNA, units and this is referred to as the L-type arrangement of rDNA (Garcia *et al.*, 2009; Garcia and Kovařík, 2013).

While there have been numerous studies investigating phylogenetic relationships between gymnosperm species by sequencing parts of the rDNA (e.g. ITSs), there is more limited understanding of how the rDNA is organized and evolves, with most studies focused on the physical organization of rDNA in conifers (especially Pinaceae) using molecular cytogenetic approaches (Lubaretz *et al.*, 1996; Brown and Carlson, 1997; Murray *et al.*, 2002; Siljak-Yakovlev *et al.*, 2002; Cai *et al.*, 2006; Miranda *et al.*, 2007; Shibata *et al.*, 2016). Nevertheless, there are a few reports of the chromosomal distribution of rDNA in *Ginkgo biloba* (Hizume *et al.*, 1999; Galián *et al.*, 2012) and *Cycas revoluta* (Hizume *et al.*, 1992; Wang *et al.*, 2016). Overall, the data indicate that these gymnosperm lineages have an S-type arrangement of rDNA, although conifers typically have multiple interstitial and pericentromeric locations of 35S rDNA (Siljak-Yakovlev *et al.*, 2002; Islam-Faridi *et al.*, 2007), whereas cycads and *Ginkgo* tend to carry 35S rDNA in subtelomeric positions (reviewed by Garcia *et al.*, 2017). In Pinaceae, the number of 5S rDNA loci has been correlated with the number of these gene families (Besendorfer *et al.*, 2005). Amongst gnetophytes, the organization of both 5S and 35S rDNAs has been studied in just three species, i.e. *Gnetum gnemon*, *Ephedra major* and *Ephedra viridis*, revealing an S-type organization in *Gnetum* and L-type organization in both *Ephedra* species (Garcia and Kovařík, 2013). An interesting property of rDNA across eukaryotes is that each unit in a tandem array is more similar, or identical, to its neighbour in the genome than might be expected by chance (Ide *et al.*, 2010). This pattern arises through concerted evolution (Zimmer *et al.*, 1980; Dover, 1982), which homogenizes the gene sequences within the rDNA arrays. The mechanism(s) responsible for this phenomenon are not completely clear, but they likely include unequal recombination and other recombination-based processes. It is widely accepted that the intragenomic homogeneity of non-coding regions of rDNA units (ITSs and IGSs) is generally lower than that of coding regions (Stage and Eickbush, 2007; Matyasek *et al.*, 2012; Weitemier *et al.*, 2015; Boutte *et al.*, 2016; Lunerova *et al.*, 2017). Nevertheless, despite the overall homogeneity of coding regions in most eukaryotic species studied to date, there are a few examples of weakly homogenized rDNA where concerted evolution seems to be ineffective. For example, (1) in *Cycas revoluta* (gymnosperm) the heterogeneity of both coding and non-coding regions was similar (high), suggesting very low frequencies of rDNA homogenization, especially in the genic regions (Wang *et al.*, 2016); and (2) in *Podisma* (grasshopper) a large fraction of the 18S rRNA genes appeared to be pseudogenized (Keller *et al.*, 2006). Elsewhere amongst the seed plants, where there is intragenomic variation in the ITS sequences this has been attributed to recent interspecific hybridization and/or polyploidy (Wei *et al.*, 2003; Campbell *et al.*, 2005; Won and Renner, 2005; Harpke and Peterson, 2006; Xiao *et al.*, 2010).

Despite a long history of cytogenetic investigations (Florin, 1932; Fernandez, 1936; Fagerlind, 1941; Mehra, 1946; Khoshoo and Ahuja, 1963), reviewed in Leitch and Leitch (2012), the genomes of gnetophytes remain largely unexplored, with the one exception of the recent report of the whole-genome sequence for *Gnetum montanum* (Wan *et al.*, 2018). To address this deficiency, we compare the structure, copy number, homogeneity and large-scale physical organization of rDNA sequences in one species from each gnetophyte genus: *Gnetum montanum* (a liana), *Ephedra altissima* (a shrub) and *Welwitschia mirabilis* (a dwarf tree). This was achieved by reconstructing the rDNA unit structures from high-throughput sequencing (HTS) data, which, in combination with fluorescent *in situ* hybridization (FISH) and Southern blot hybridization, has revealed an astonishing diversity that is unparalleled in other gymnosperm lineages.

## MATERIALS AND METHODS

### Plant material and genomic DNA extraction

Fresh leaf material from *Welwitschia mirabilis* (accession number 441-05-9175) and fresh stems of *Ephedra altissima* (accession number 1991-0351, WW-EA-2013 voucher specimen QMUL_EA_11-17) were obtained from the Chelsea Physic Garden, London, UK, while fresh leaves of *Gnetum montanum* (accession number XHMMT01) were collected from the Shenzhen Fairy Lake Botanical Garden, Shenzhen, China. In addition, fresh leaf material of *Gnetum gnemon* was collected from the Royal Botanic Gardens, Kew (accession number 1998-514). All fresh material was dried in silica gel for later use. Genomic DNA was extracted using the CTAB method (Doyle, 1991). The quality and quantity of total genomic DNA were evaluated by NanoDrop (Thermo Scientific, USA) and a Qubit 2.0 fluorometer (Life Technologies, USA).

### Illumina HiSeq sequencing and sequence resources

Total genomic DNAs from *E. altissima* and *W. mirabilis* were sequenced at Beijing Genome Institute (BGI), Shenzhen, China using an Illumina HiSeq 2000 platform (170-bp library insert size, 90 bp read length). The sequencing of *G. montanum* (paired-end) was carried out at Novogene, Beijing, China, using an Illumina HiSeq 2000 platform (300-bp library insert size, 100 bp read length). FASTQ format sequencing reads were supplied with adapter sequences removed. A summary of the sequencing information is available in Table 1.

**Table 1.** Genome size (1C value) and volume of Illumina HiSeq paired-end (PE) reads analysed and genome proportion (GP) for the three gnetophyte species analysed with RepeatExplorer

Species	Genome size (Gb)	No. of paired-end reads analysed for clustering	Genome sequencing depth	Read length (bp)	GenBank accession number
*Ephedra altissima*	18.5^a^	10 296 111	0.05×	90	ERR845261/ ERS1497378
*Gnetum montanum*	4.2^b^	2 000 000	0.05×	100	ERS1497380/ERS1497379
*Welwitschia mirabilis*	7.2^c^	6 960 348	0.087×	90	ERR845262/ ERS1497381

^a^Data from this study.

^b^Data from Wan *et al.* (2018).

^c^Data from the Plant DNA C-values Database (Bennett and Leitch, 2012).

### Assembling rDNA units and 5S rDNA sequence alignment

For repetitive sequence analysis, Illumina read volumes equivalent to ~5–9 % of the genome of *E. altissima*, *W. mirabilis* and *G. montanum* were imported into the RepeatExplorer (RE) software (Novak *et al.*, 2013), a pipeline developed for the analysis of repetitive DNA in genomes using HTS data and implemented in the Galaxy environment (Table 1). Poor-quality reads, with a Phred score of <20 for >10 % of their bases, were removed using tools embedded in RE and analysed individually. Using all-to-all BLAST, RE displays read similarities in the form of a graph in which reads are represented as nodes and reads that share at least 90 % similarity over at least 55 % of their length are connected by lines that increase in length with decreasing sequence similarity. The graph containing all input reads is then split into clusters as described in Novak *et al.* (2010). For each of these clusters RE supplies one or more contigs assembled from the cluster’s reads. While clusters often represent specific repeat types, repeats with a large unit length, such as rDNA sequences, whose units often exceed 10 kb (Hemleben *et al.*, 1988; Bobola *et al.*, 1992), are often fragmented into multiple clusters, as observed here. A detailed investigation of clusters with similarity to known rDNA sequences allowed concatenation to produce contigs (termed HTP contigs) using paired-end read sequence data (cluster merger tool in RE). Additionally, HTP contigs were concatenated manually after visual inspection of pairwise alignments.

Sequence alignments for 5S rDNA sequences (which are typically much shorter than 35S rDNA units), including 5S coding and pseudogenized sequences, were conducted using Geneious 10.1 (Biomatters, New Zealand) with default settings. The sequences from the three studied species were aligned against the 5S rRNA gene from *Ephedra kokanica* (GenBank accession number X06996).

Sequence alignments for S-type (*G. montanum* and *W. mirabilis*) and L-type (*E. altissima*) rDNA sequence organizations were also carried out using Geneious 10.1 (Biomatters, New Zealand) with default settings. A phylogeny nearest neighbour tree was constructed from aligned ITS1 sequences using an online program (Dereeper *et al.*, 2008).

### Single-nucleotide polymorphism and copy number analysis

The CLC Genomics Workbench 7.1. (QIAGEN, https://www.qiagenbioinformatics.com/) was used to estimate intragenomic variation between multiple rDNA units. The input reads from RE were first mapped to the rDNA contigs (obtained from RE) with the mapping settings as in Wang *et al.* (2016). Only high-confidence single-nucleotide polymorphisms (SNPs) occurring at a frequency of ≥20 % were considered. The distribution of SNPs along the units was diagrammatically depicted using R software (R Core Development Team, 2013). SNP counts and copy numbers were determined using total Illumina reads (typically >7 million) mapped to rDNA reference sequences (from HTP consensus) trimmed for most of the IGSs. SNPs were called in mapped reads with parameter settings of at least 40 identical SNPs in at least 200 reads (with the exception of *W. mirabilis*, where a threshold of 100 reads was used due to low coverage). An SNP was recorded when it occurred in ≥20 % of reads. The SNP frequency in individual subregions of rDNA was expressed as the number of SNPs per unit length of DNA (1 kb). The genome proportion and copy number of 18S and 5S RNA genes were calculated from the number of mapped reads out of total reads according to procedures described in Supplementary Data Table S1. The 18S rRNA genes were more evenly covered than the other regions and hence were preferentially used for copy number estimation.

### rDNA cluster homo/heterogeneity analysis

All-to-all BLAST searches (*e* values ≤10^−5^) were carried out using reads in each rDNA cluster and the pairwise similarities recorded. Reciprocal hits and self-hits were filtered from the BLAST results with a customised Perl script (available on request). Histograms of the percentage similarities between reads from each of the rDNA clusters were generated in R (R Core Development Team, 2013).

### FISH

Freshly harvested root tips of *W. mirabilis* were collected from young plants (pot plants <10 years old) growing in pots at the Royal Botanic Gardens, Kew (Richmond, UK). Root tips were immersed in a saturated solution of α-bromonaphthalene for ~2 h at room temperature (RT). Freshly harvested root tips from a mature, wild-growing tree of *G. montanum* were collected from Shenzhen Fairy Lake Garden (Shenzhen, China). Root tips were immersed in 0.05 % (w/v) colchicine for ~2 h at RT. Fresh shoot tips, including one or two stem nodes of *E. altissima*, were collected from an adult pot-plant growing at Queen Mary University of London (London, UK) before being pretreated with 0.02 m 8-hydroxyquinoline at RT for ~4 h.

Following pretreatment, all root and shoot tips were fixed in freshly prepared 3:1 (v/v) ethanol:glacial acetic acid overnight or for 24 h at RT before transfer to 70 % (v/v) ethanol and stored at −20 °C until use. Fixed root tips and shoots were incubated in an enzyme solution [1 % (v/v) pectinase and 2 % (v/v) cellulase in citrate buffer] for 90–120 min at 37 °C, as described in Becher *et al.* (2014). After enzyme digestion, root tips of *W. mirabilis* and *G. montanum* and the meristem tissues around the node of *E. altissima* shoot tips were gently cut into fragments using a dissecting knife and needle under a Leica stereo microscope (Leica Microsystems, Germany) and washed using 65 % (v/v) acetic acid before squashing the digested cells onto a glass slide.

The 18S and 5S rDNA probes were labelled with Alexa Fluor 488 (Thermo Fisher Scientific, USA) and Texas Red (Thermo Fisher Scientific, USA), respectively, as described in Becher *et al.* (2014). Hybridization was carried out according to standard procedures as described in Schwarzacher and Heslop-Harrison (2000). FISH signals were visualized using an Olympus AX 70 fluorescence microscope equipped with a digital camera. Images were analysed and processed using ISIS software (MetaSystems, Altlussheim, Germany). For each species >20 metaphases with signals were examined.

### Prediction of the secondary structure of 5S rRNA

The secondary structures of the 5S rRNA sequences [extracted from HTP contigs generated here or from GenBank clones for *G. gnemon* (GenBank accession number EU882731) and *E. major* (GenBank accession number JX843794)] were examined to predict their likely functionality. The structures were computed based on minimum free energy values and the partition function of the calculation algorithm. All calculations were carried out using the RNAfold WebServer [http://RNA.tbi.univie.ac.at (Lorenz *et al.*, 2011)]. Isolated base pairs were avoided.

### Southern blot hybridization

Most DNAs for the Southern analysis were identical with those used for whole-genome sequencing; *G. gnemon* DNA was isolated from an individual in the Royal Botanic Gardens, Kew (Richmond, UK). About 4 μg of purified *G. montanum*, *G. gnemon*, *W. mirabilis* and *E. altissima* DNAs were digested with BamHI and BstNI restriction enzymes. The genomic DNAs of *E. altissima* were also digested using EcoRI, NcoI and StuI to reveal the linkage between 5S and 35S rRNA genes. The digested genomic DNAs were separated by agarose gel (0.9 % w/v) electrophoresis. The DNA fragments were then blotted onto Hybond-XL membranes (GE Healthcare, Little Chalfont, UK) and hybridized with the ^32^P-labelled 5S and 18S rDNA probes as described in Garcia and Kovařík (2013) following protocols outlined in Kovařík *et al.* (2000). Membranes were washed under high-stringency conditions as described in Wang *et al.* (2016) and scanned with a PhosphorImager (Typhoon 9410, GE Healthcare, PA, USA), and the signals were analysed using ImageQuant software (GE Healthcare, PA, USA). Slot-blot hybridization was carried out with 100, 50 and 25 ng of genomic DNA blotted onto a Hybond-XL membranes using a 24 × 3 slot apparatus (Schleicher & Schuell, Germany). Hybridization conditions and the probes were as above.

## RESULTS

### Reconstruction of rDNA units from Illumina HiSeq data and estimation of rDNA copy numbers

**Fig. 1.**
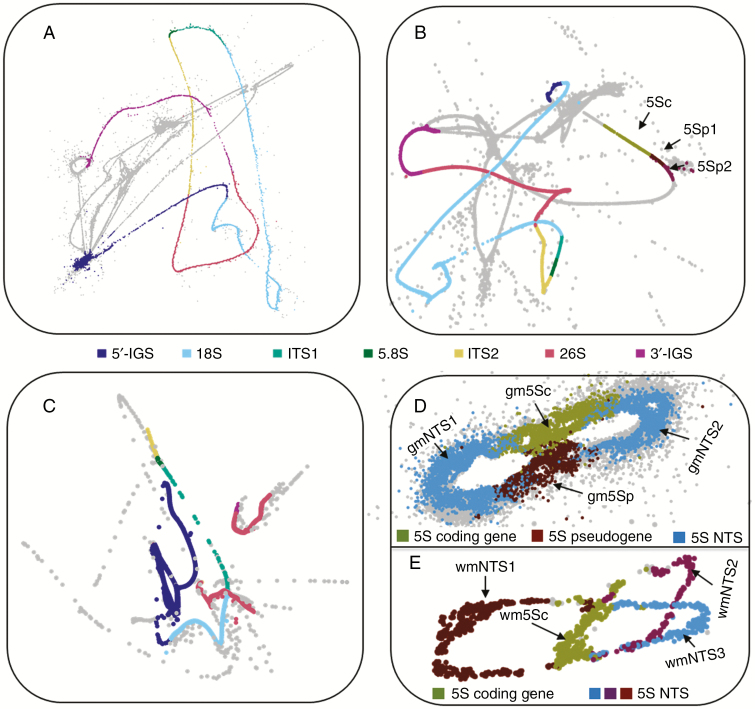
Projections of RepeatExplorer graphs representing rDNA units in gnetophytes. Graphs show rDNA paths in (A) *G. montanum* 35S rDNA, (B) *E. altissima* linked 35S–5S rDNA, (C) *W. mirabilis* 35S rDNA, (D) *G. montanum* 5S rDNA and (E) *W. mirabilis* 5S rDNA. Individual subregions of 35S rDNA are highlighted (see legend in the centre of the figure). The 5S subregions indicated are as follows: (B) 5Sp1 and 5Sp2 correspond to 5S pseudogene 1 and 2, respectively; (D) NTS, non-transcribed spacer (region); 5Sc, 5S rDNA coding sequence; 5Sp, 5S rDNA pseudogene; (E) NTS1, NTS2, NTS3, putative NTS categories 1, 2 and 3; 5Sc, 5S rDNA coding sequence.

Low-coverage Illumina HiSeq genomic sequencing data of *E. altissima*, *G. montanum* and *W. mirabilis* (Table 1) were analysed using RE. The graphs of RE clusters comprising reads with sequence similarity to rDNA (rDNA clusters) are shown in Fig. 1. In *G. montanum* and *W. mirabilis* there were clusters that showed similarity to 18S–5.8S–26S genes (35S rDNA) and others that showed similarity to 5S rRNA genes (5S rDNA), indicating that both *G. montanum* (Fig. 1A, D) and *W. mirabilis* (Fig. 1C, E) have an S-type arrangement of rDNA. In contrast, RE grouped all four rRNA genes (and pseudogenes; see later) of *E. altissima* into a single cluster in the following order: 18S–5.8S–26S–5S rDNA (Fig. 1B). The interrupted 35S rDNA RE graph of *W. mirabilis* is best explained by low coverage of that region rather than the insertion of non-rDNA sequences or sequencing errors.

Reads in clusters from the 35S rDNA for *E. altissima*, *G. montanum* and *W. mirabilis* and the 5S rDNA for *E. altissima*, and *W. mirabilis* formed smooth lines in the graphical displays (Fig. 1A–C, E), indicating little sequence variation between these multiple rDNA units. In contrast, the output graph of the 5S rDNA cluster for *G. montanum* (Fig. 1D) was more diffuse, indicative of more divergent reads.

Mapping of reads to assembled 18S and 5S rDNA subregions, together with genome size information and the estimated lengths of the rRNA genes for each species, enabled the copy numbers and genome proportion (GP) occupied by the different rRNA genes to be estimated (Supplementary Data Table S1). The data revealed that the copy numbers of the 18S (equivalent to 35S) rDNAs had a >2-fold range (2500–6000 copies), while 5S rDNA copy numbers had a 27-fold range (3900–105 200 copies) (Table 2). The highest copy numbers for the 5S rDNA were found in *G. montanum*, which had seven times more copies than *E. altissima* and 27 times more than *W. mirabilis*. Experimental verification of copy numbers was carried out using slot-blot hybridization (Table 2). The 5S rDNA copy numbers determined by conventional slot-blot hybridization were always lower than those calculated *in silico* (Table 2). This may be explained by a failure of probe hybridization to mutated copies and pseudogenes. The experimentally determined 18S rDNA copy numbers were congruent with the calculated numbers. In *W. mirabilis* the hybridization signals were weak and could not be quantified.

**Table 2.** Information on chromosome numbers (2n) and rDNA characterization in the three gnetophyte species analysed

Species	No. of chromosomes (2*n*)	No. of 18S/5S rDNA sites at metaphase^a^	Estimated length of 35S/5S rDNA contigs (bp)	Estimated GP (%)/CN of 18S rDNA^b^		Estimated GP (%)/CN of 5S rDNA^b^	
				Calculated from HTP^c^	Slot-blot hybridization^d^	Calculated from HTP^c^	Slot-blot hybridization^d^
*Ephedra altissima*	28^e^	14–16	6766	0.058/6000	0.060/6200	0.010/15000	0.005/7700
*Gnetum montanum*	44^f^	~6–8/>10	6527/627	0.132/3100	0.142/4000	0.301/105200	0.250/87400
*Welwitschia mirabilis*	42^g^	2/2	5981/958	0.063/2500	n.d.^h^	0.007/3800	n.d.^h^

^a^Site numbers are based on FISH (Fig. 5).

^b^GP and CN, genome proportion and copy number of rDNA per 1C.

^c^GP estimated by (the number of mapped reads) / (total number of reads analysed), CN was estimated by (GP×genome size) / (estimated rDNA sequence length) (Supplementary Data Table S1), the reference sequences were 120 bp of 5S and c.1800 bp of 18S rRNA genes from the assembled RE contigs.

^d^GP and CN were estimated experimentally from slot-blot hybridization signals (Supplementary Data Fig. S10).

^e^ Resende (1936) and this study.

^f^ Wan *et al.* (2018) and this study.

^g^ Florin (1932) and this study.

^h^n.d. not determined.

### 35S rDNA arrangement and structure in gnetophytes

**Fig. 2.**
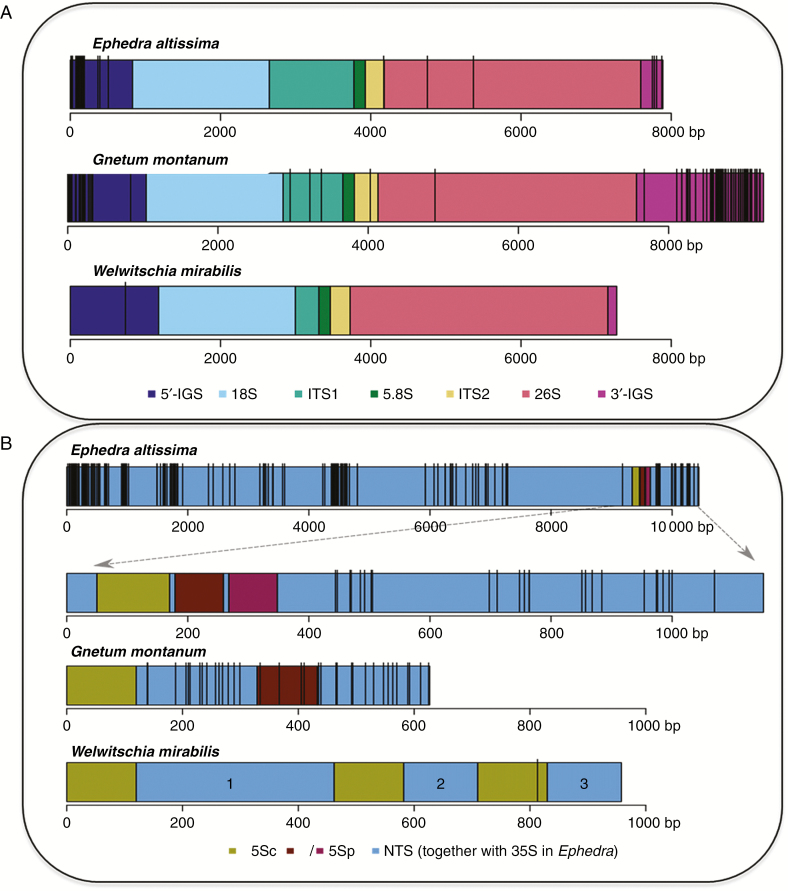
Distribution of SNPs across the 35S and 5S rDNA sequences in *E. altissima*, *G. montanum* and *W. mirabilis*. The identity of the rDNA unit subregions is shown by their colour. Vertical black bars represent high-confidence (onf % frequency) SNPs in (A) 35S rDNA and (B) 5S rDNA. In (B) the *E. altissima* IGS subregion is enlarged (dashed arrows) to show the organization of three integrated 5S rDNA sequences, including the functional 5S gene (5Sc) and the two 5S pseudogenes (5Sp).

Pairwise comparison of all contigs carrying rDNA sequences provided sufficient overlap to construct a consensus sequence for the whole 18S–5.8S–26S rDNA array in each species (Fig. 2 and Supplementary Data Fig. S1A). The total length of the 18S–5.8S–26S rDNA unit (excluding the IGS) ranged from 5981 bp in *W. mirabilis* to 6527 bp in *G. montanum* and 6766 bp in *E. altissima*, with variation arising predominantly from length differences of the ITS1 region (Supplementary Data Fig. S2A). The longest ITS1 was in *E*. *altissima* (1160 bp), which contained 2.8 copies of a 71-bp GC-rich (73 %) repeat close to its 3′ terminus (Supplementary Data Fig. S3A). In contrast, ITS1 was relatively short in *W. mirabilis* (313 bp) and *G. montanum* (796 bp) and in both it lacked repetitive elements (Supplementary Data Fig. S2A). The ITS1 subrepeats in *E*. *altissima* were highly conserved across the genus (Supplementary Data Fig. S4 and Table S2). Nevertheless, despite such an overall structural conservation between species, the divergence of ITS1 allowed us to build a gene tree from the GenBank clones and the *E. altissima* HTS contig (Supplementary Data Fig. S5). On the phylogram, the Old and New World species formed well-supported sister clades, with *E. altissima* forming part of the Old World branch.

We were unable to reconstruct the complete 35S rDNA units, which include the whole of the IGS, in any of the species, probably due to the repetitive nature of the IGS and its large size (Bobola *et al.*, 1992). Relatively large parts of the IGS were retrieved in *E. altissima*, where abundant minisatellites were detected in regions flanking the 5S rDNA insertions (Supplementary Data Fig. S3).

### 5S rDNA arrangement and structure in gnetophytes

An RE cluster from *W. mirabilis* carrying 5S rRNA gene sequences yielded an HTS contig that had three 120-bp 5S rRNA gene copies (wm5Sc), each associated with a unique NTS spacer (wmNTS1, wmNTS2, wmNTS3) (Figs 1E and 2 and Supplementary Data Fig. S6). This arrangement formed three loops in the graphical output of RE, each including a putative functional gene (Fig. 1E). The three putative 5S rRNA genes have diverged from each other, although their overall similarity to the sequenced cytosolic 5S rRNA from *E. kokanica* (Melekhovets *et al.*, 1988) (GenBank accession number X06996) remained high (Supplementary Data Fig. S1B). The 2-D structure of their putative transcripts (Supplementary Data Fig. S7) was also well conserved, indicating functionality. The wmNTS1 spacer is 342 bp in length (dark red loop in Fig. 1E and NTS domain 1 in blue in Fig. 2B). The wmNTS2 and wmNTS3 spacers were 128 bp (blue and purple loops in Fig. 1E and NTS domains 2 and 3 in blue in Fig. 2B). The long wmNTS1 spacer differed from its short wmNTS2 and wmNTS3 versions by two insertions of 120 and 95 bp, respectively (Supplementary Data Fig. S6). The insertions were 63 % identical and did not match any sequence in the GenBank.

In *G. montanum* an HTS contig had two putative 5S rRNA genes (Figs 1D and 2B). The first copy (labelled gm5Sc) was 120 bp long. It showed a high sequence similarity to other 5S rRNA genes in GenBank (Supplementary Data Fig. S1B) and reconstructions of secondary structures indicated high thermodynamic stability of its rRNA (Supplementary Data Fig. S7). The second 5S rDNA copy (termed gm5Sp) was only 104 bp in length. This copy had relatively low (83 %) sequence similarity to gm5Sc and other 5S genes (Supplementary Data Fig. S1B) and its putative transcript exhibited a distinct secondary structure (Supplementary Data Fig. S7). The 5S rDNA tandems were separated by two NTS regions (NTS1 is 209 bp, NTS2 is 194 bp). In *E. altissima* an HTP contig was recovered that harboured three putative 5S rDNA copies (Fig. 2B). The 120 bp-long copy, located proximal to the 18S gene, had high similarity to other functional 5S genes (Supplementary Data Fig. S1B). The two neighbouring 5S copies each had a 40-bp deletion at the 5′ end. Their similarity to functional 5S rRNA and to the first copy was low. The three 5S rDNA copies, one functional (em5Sc) and two that are likely to be pseudogenes (em5Sp1 and em5Sp2), were separated by two unusually short (9 bp) NTSs (ATTTTTATC and CATTTTATC). Many *E. altissima* reads mapped to the 5S–18S clone from *E. major* (GenBank accession number JX843794). The IGS of *E. altissima* showed high structural similarity to the *E. major* clone with respect to the arrangement, direction of transcription, presence of short non-transcribed spacers and deletions in the 5′ ends of the pseudogenes (Supplementary Data Figs S1B and S7).

### Southern blot hybridization reveals the genomic organization of rDNA

**Fig. 3.**
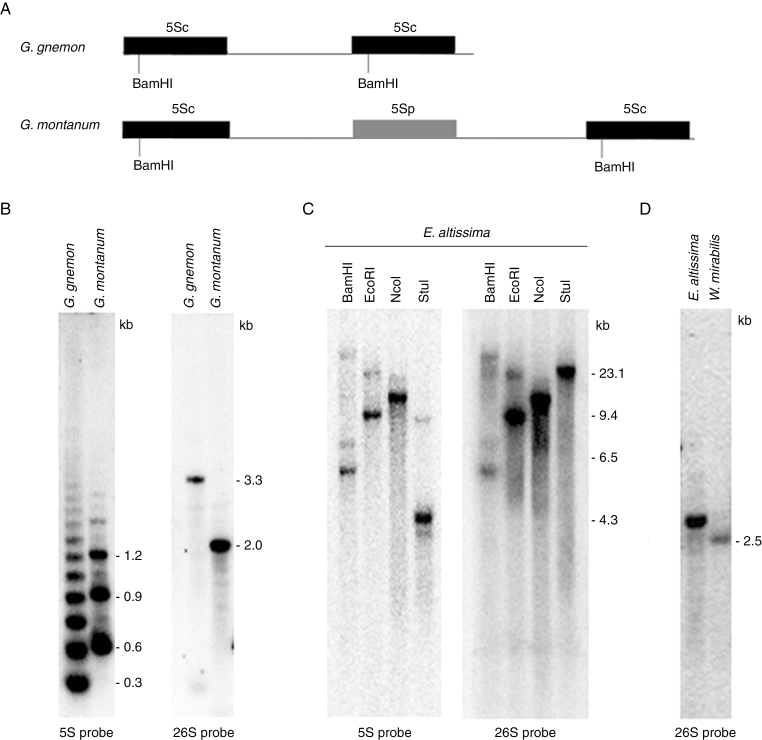
Southern blot hybridization analysis showing the genomic organization of the 5S and 35 rDNA regions in *E. altissima*, *G. montanum* and *W. mirabilis*. (A) Restriction maps of 5S units in G. *montanum* HTS contig and *G. gnemon* clone (GenBank accession number EU882731). Conserved BamHI sites in functional 5S genes (5Sc) are indicated. (B) Hybridization of BamHI (5S rDNA probe)- and BstNI (26S rDNA probe)-digested genomic DNA of *G. montanum* and *G. gnemon*. Note that the probes hybridize to restriction fragments of different size and that the periodicity of BamHI-generated ladders with the 5S probe differs between the two species. (C) A similar hybridization experiment to size fractionated *E. altissima* genomic DNA digested with BamHI, EcoRI, NcoI and StuI restriction enzymes. Note the co-hybridization of 5S and then 26S rDNA probes to the same BamHI, EcoRI and NcoI restriction fragments. (D) 26S probe hybridization to *E. altissima* and *W. mirabilis* DNA digested with BstNI. Note the very weak signal in *W. mirabilis.*

To verify the predicted arrangement of rDNAs, we carried out Southern blot hybridization using 26S and 5S rRNA gene probes. Southern blot hybridization of *G. montanum* and *G. gnemon* 5S rDNA revealed a ladder of evenly spaced BamHI fragments, indicating a tandem arrangement of units (Fig. 3B). The size of the *G. montanum* monomer (~0.7 kb) was consistent with the restriction map of its assembled unit (Figs 2B and 3A), which suggested that the NTS-embedded pseudogene (gm5Sp) lacked a BamHI site (Supplementary Data Fig. S1B). Functional copies of 5S rRNA genes are thought to contain BamHI sites in the first half of their genes (Roser *et al.*, 2001). The size of the *G. gnemon* monomer (~0.3 kb) corresponded well to the size predicted from the GenBank clone sequence (accession number EU882731) (Fig. 3A). The hybridization pattern of the 26S probe differed between *G. montanum* and *G. gnemon*, most probably reflecting IGS polymorphisms between these species.

The BamHI, EcoRI, NcoI digestion of *E. altissima* genomic DNA revealed that both 5S and 26S rDNA probes co-hybridized to common restriction fragments (Fig. 3C), further confirming a linked arrangement of 35S and 5S rRNA genes in this species. The 26S rDNA probe hybridized to a ~22-kb StuI fragment while the 5S rDNA probe hybridized to a ~4-kb fragment. This can be explained by the presence of a StuI site(s) in the intergenic spacer between the 26S and 5S rRNA genes. The sum of fragments indicated that the size of the *E. altissima* rDNA unit (including the IGS) was at least 26 kb.

In *W. mirabilis*, the 26S rDNA probe hybridized to a single ~2.5-kb fragment (Fig. 3D) much more weakly than in other species. We were unable to detect the 5S rDNA probe hybridization to *W. mirabilis* DNA, probably because of sensitivity limitations of Southern blot approaches (there are relatively few copies of both 26S and 5S rDNA in its large genome; Table 2).

### Variable intragenomic homogeneity of rDNA in gnetophytes

Two approaches were used to determine the homogeneity of rDNA repeats in gnetophytes.

**Fig. 4.**
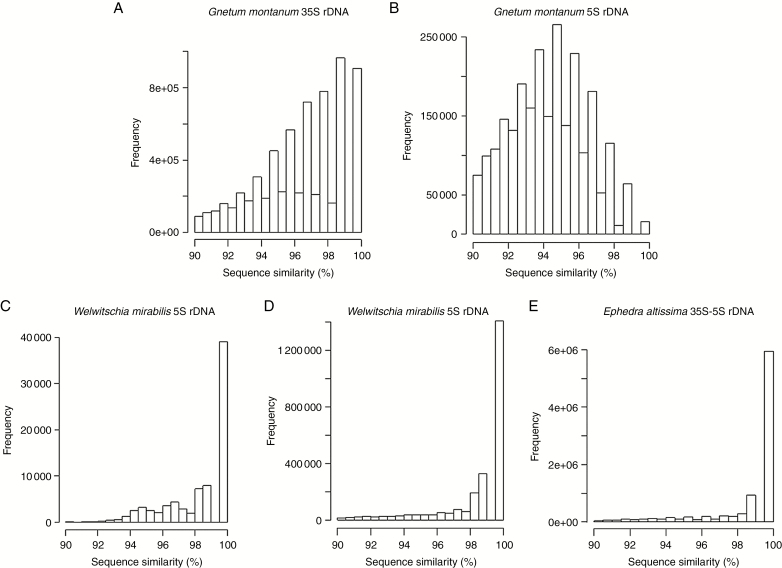
Read sequence heterogeneity of rDNA in gnetophytes. Histograms showing the percentage sequence similarity between reads from 35S (A, D), 5S (B, C) and 35S–5S (E) rDNA clusters in *G. montanum* (A, B), *W. mirabilis* (C, D) and *E. altissima* (E).

(1) The abundance of SNPs in assembled rDNA units (based on a threshold of SNPs occurring in ≥20 % reads – see the Materials and methods section) was analysed in the different subregions of both the 35S and 5S rDNA units (Fig. 2 and Supplementary Data Fig. S8). In *E. altissima* SNPs were predominantly located in the IGS while surprisingly there were no SNPs in the pseudogenized 5S rDNA copies. Similarly, in *G. montanum* the SNP abundance was high in IGS. In contrast to *E. altissima*, *G. montanum* showed unusually high SNP abundance (up to 88 SNPs kb^−1^) in 5S rDNA pseudogenes and NTS regions (Fig. 2B, Supplementary Data Fig. S8 and Supplementary Data Table S3). Out of the three species, *W. mirabilis* displayed the lowest SNPs frequency in both 5S and 35S rDNA units (Fig. 2 and Supplementary Data Fig. S8).
(2) All-to-all BLAST searches of reads within the clusters generated by RE were used to generate histograms to show the percentage sequence similarity between read pairs in each rDNA cluster (Fig. 4). The histograms revealed larger sequence diversity between reads in both the 5S and 35S rDNA clusters of *G. montanum* compared with reads from *E. altissima* and *G. montanum*. Yet despite this greater diversity of reads in *G. montanum*, some reads with high similarities do occur, albeit in relatively low abundance (Fig. 4A, B).

Overall, both approaches revealed high intragenomic diversity of 5S rDNA in *G. montanum* compared with the relatively homogeneous corresponding arrays in *E. altissima* and *W. mirabilis*, a result also consistent with the graphical displays of RE (Fig. 1), which show a more diffuse cluster in *G. montanum* (Fig. 1D) compared with those of the other two species (Fig. 1B, E).

### FISH

**Fig. 5.**
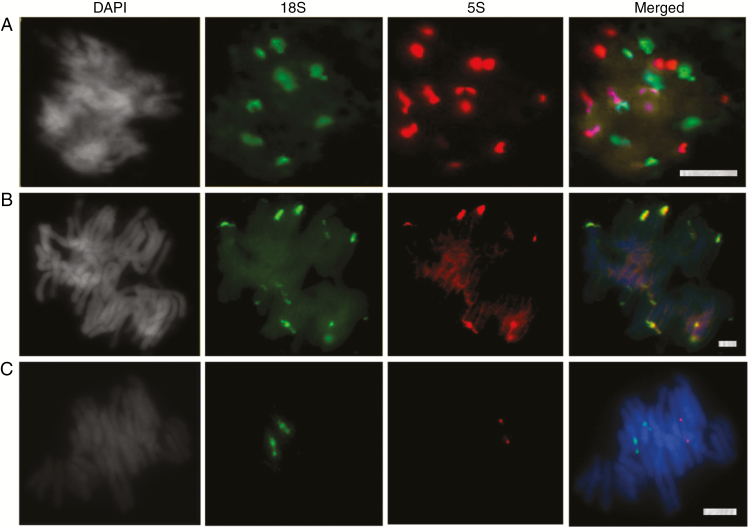
Fluorescent *in situ* hybridization of rDNA probes to: (A) *G. montanum*, (B) *E. altissima* and (C) *W. mirabilis*. Signals of 18S and 5S rDNA probes are in green and red, respectively. DAPI staining for DNA appears in grey to enable the visualization of chromosome contours. Scale bars = 10 µm.

Obtaining high-quality metaphase spreads from any of the species proved to be extraordinarily difficult, which is why we do not present karyotypes here. This was particularly true for the root tips of the wild-growing *G. montanum* [a species whose genome has been sequenced and assembled (Wan *et al.*, 2018)]. Nevertheless, in *G. montanum* the 18S rDNA probe was seen to hybridize to between six and eight sites on the *G. montanum* chromosomes while the 5S rDNA probe hybridized to at least ten sites. None of the sites showed co-hybridization of probe signals, providing supporting evidence for an S-type arrangement of rDNA loci in this species (Fig. 5A).

In the metaphases of *E. altissima*, the 18S rDNA probe hybridized to 14–16 sites; of these, two labelled strongly, six had intermediate label strength and six to eight were minor (Fig. 5B). All 5S rDNA probe hybridization sites overlapped with the major 18S rDNA sites (Fig. 5B), providing supporting evidence of an L-type arrangement. The absence of 5S rDNA signals co-localizing with the minor 18S rDNA sites is most likely due to the weaker hybridization of the 5S probe. All signals were in subtelomeric positions.

In the metaphases of *W. mirabilis* (Fig. 5C), the 18S and 5S rDNA probes hybridized to different chromosomes (two sites of each), indicating a separate arrangement. The 5S rDNA sites appeared to be highly condensed, whilst the 18S rDNA was partially decondensed, exhibiting secondary constriction on both homologues (Fig. 5C).

## DISCUSSION

The four extant gymnosperm lineages, i.e. cycads, *Ginkgo*, conifers and gnetophytes, are thought to have diverged during the Late Carboniferous to the Late Triassic (Wang and Ran, 2014). However, as noted in the Introduction, there remains controversy over the precise phylogenetic placement of gnetophytes in relation to other seed plants, although the current emerging consensus suggests they form a clade that is sister to, or within, the conifers (Wickett *et al.*, 2014), perhaps diverging from remaining conifers ~150 MYA (Lu *et al.*, 2014; Li *et al.*, 2017).

### ITS lengths in gnetophytes

The length of ITS1 is generally greater in gymnosperms compared with angiosperms due to the presence of repeated elements (Maggini and Baldassini, 1995; Liston *et al.*, 1996; Gernandt *et al.*, 2001; Ickert-Bond and Wojciechowski, 2004). In Pinaceae, the ITS (ITS1–5.8S–ITS2) subregion has been reported to range between 1550 and 3125 bp (Liston *et al.*, 1996; Puizina *et al.*, 2008). Amongst the gnetophytes, only *E. altissima* harboured ITS of comparable length (1530 bp), and it too carried repetitive elements that showed no similarity to those found in Pinaceae. The subrepeated region of ITS1 was relatively short in *Ephedra*, accounting for <20 % of ITS1, while in *Pinus* the subrepeats formed a major (>90 %) part of ITS1 (Supplementary Data Fig. S9). Further analysis showed that both the length of ITS1 and its repeat composition were highly conserved across *Ephedra* species, suggesting that this subregion has changed little during the radiation of the extant species, which is estimated to have started around 30 MYA during the Oligocene (Ickert-Bond *et al.*, 2009). This is surprising given that the tandem subrepeats tend to diverge rapidly in rDNA IGSs (Sano and Sano, 1990; Volkov *et al.*, 1999; Carvalho *et al.*, 2011; Lunerová, 2017). In *G. montanum* and *W. mirabilis* the ITS1 subregions were significantly smaller (1150 and 650 bp, respectively) and lacked detectable subrepeats, which is the situation also found in the ITS1 of angiosperms. Thus, ITS1 subregions of gnetophytes exhibit both angiosperm-like (*G. montanum* and *W. mirabilis*) and gymnosperm-like (*E. altissima*) features.

In contrast to ITS1, the lengths of ITS2 subregions in gnetophytes were similar to those in other gymnosperms and angiosperms that have been studied (Liston *et al.*, 1996). These data may suggest strong selection pressures for the maintenance of a uniform ITS2 length in seed plants. Perhaps the maturation of 35S preRNA transcripts into functional rRNAs requires a uniform, non-repetitive structure of ITS2, whilst requirements for ITS1 structure are more relaxed. Tandem repeats were not detected in any assembled 5S units of the gnetophytes analysed, whilst they have been reported in the NTS regions of Pinaceae 5S rDNA (Besendorfer *et al.*, 2005).

### Divergence (or lack of it) in the organization of rDNA loci

**Fig. 6.**
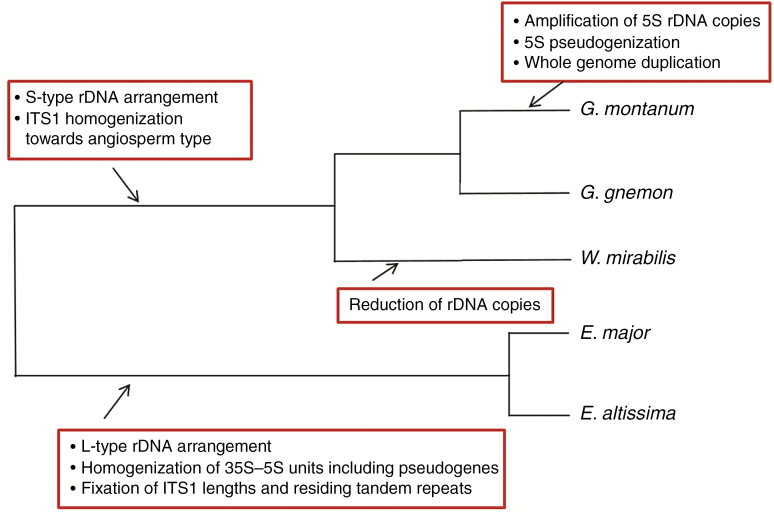
A phylogenetic tree depicting the evolutionary relationship between gnetophyte species with the branch lengths approximating to the degree of sequence divergence [based on nuclear (ITS) and chloroplast markers] between species (Hou *et al.*, 2015; Ickert-Bond and Renner, 2016). Assuming this topology, the predicted patterns of divergence in the evolution of rDNA of gnetophytes are described.

Two types of rDNA arrangement have been identified in gnetophytes: (1) the S-type arrangement of 35S and 5S rRNA genes has been found in *W. mirabilis* and both *Gnetum* species (*G. montanum* and *G. gnemon*); and (2) the L-type arrangement of 35S–5S rDNA units has been found in *E. altissima* (this work), *E. major* and *E. viridis* (Garcia and Kovařík, 2013). In *E. altissima*, FISH did not reveal any non-overlapping 5S and 35S rDNA signals and all seven or eight loci (per haploid set) were located distally. A subtelomeric position of rDNA was previously identified in *Ephedra americana* (Hizume and Tominanga, 2016), although that study, which was based on chromomycin A3/DAPI (4′,6-diamidino-2-phenylindole) banding methods, was not able to confirm that the 5S and 35S rDNA were co-localized. Phylogenetic studies reveal that *E. altissima* and *E. major* occur in a clade of Old World species, whilst *E. viridis* and *E. americana* belong to the clade comprising New World species (Ickert-Bond and Wojciechowski, 2004; Ickert-Bond *et al.*, 2009; Loera *et al.*, 2015) (Supplementary Data Fig. S5). The occurrence of the L-type arrangement in species from both these clades suggests that the arrangement arose in their common ancestor before the split, estimated to be around 30 MYA (Ickert-Bond *et al.*, 2009; Ickert-Bond and Renner, 2016) (Fig. 6). Thus, the hypothesis that the L-type arrangement represents a transient unstable organization of rDNA (Garcia *et al.*, 2009) is unsupported in *Ephedra*. Nevertheless, such previous assumptions were mostly based on the observation that a linked organization of 35S–5S rDNA units was rarely found in angiosperms whilst in gymnosperms (Garcia and Kovařík, 2013) and early-diverging land plant lineages, such as bryophytes (Wicke *et al.*, 2011), it is quite common.

Available fossil records suggest that *Ephedra* diverged from other gnetophytes in the Cretaceous (Rydin *et al.*, 2004; Ickert-Bond and Renner, 2016). Subsequent species radiation has given rise to species with few changes in vegetative and reproductive structures. The conservation of rDNA units associated (together with conservation in the ITS1 repeat composition – see below) with species divergence suggests a genome structure that has changed little, despite the reported high frequency of polyploidy in *Ephedra* (~37 polyploid species making up ~70 % of total extant species) (Ickert-Bond and Renner 2016; S. Ickert-Bond *et al*., University of Alaska, USA, pers. comm.), a feature that is absent or rare in other gymnosperms. However, there is currently no evidence to suggest that these polyploidy events are associated with extensive genomic, epigenomic and metabolomic changes (e.g. sub- and neo-functionalization of genes, genome restructuring and downsizing, and chromosomal rearrangements) leading to genome diploidization, as reported in angiosperms (Wendel, 2015; Van De Peer *et al.*, 2017; S. Ickert-Bond *et al*., University of Alaska, USA, unpubl. res.). Further genome-wide analyses are clearly needed to test this hypothesis.

Both *G. gnemon* and *G. montanum* harbour tandem organizations of 5S rDNA units, whilst a pseudogenized copy was found in the NTS of *G. montanum* only. The lineage leading to *G. gnemon* diverged relatively early [around the Cretaceous/Tertiary boundary, ~65 MYA (Hou *et al.*, 2015)] from the rest of the genus, whilst *G. montanum* occurs in a lineage that diverged from sister species more recently (Hou, 2016). Since patterns of rDNA sequence divergence in gymnosperms can be unusual, as noted in *Cycas*, where a low frequency of homogenization of rDNA was reported (Wang *et al.*, 2016), the ancestral condition of rDNA (i.e. 5S rDNA units with or without pseudogenes) is difficult to assess. Two 5S rDNA families have also been detected in the conifer *Abies alba* (Besendorfer *et al.*, 2005). It will be interesting to determine their functional status given the overall high content of pseudogenes in conifer genomes (Nystedt *et al.*, 2013).

### Variable rDNA sequence homogeneity in gnetophytes

Homogeneous arrays of 35S rDNA are typical of angiosperms, and this contrasts with what has been reported in some cycads (the only gymnosperm lineage to be analysed in detail so far), where both 35S (Wang *et al.*, 2016) and 5S rDNA loci (W. Wang *et al*., unpubl. res.) exhibit extraordinary sequence diversity. We were interested to determine whether such heterogeneity also occurs in gnetophytes. In contrast to cycads, across all gnetophytes examined the sequence homogeneity of 35S rDNA coding regions was high, irrespective of copy and locus numbers, suggesting efficient concerted evolution, as reported in most other eukaryotic species, including conifers and *Ginkgo*.

The non-coding regions are typically expected to display higher intragenomic heterogeneity than coding regions, especially in species with large numbers of rDNA copies (Eickbush and Eickbush, 2007). In conifers, the high divergence of NTS was attributed to slow homogenization following speciation (Liu *et al.*, 2003). Given the large differences in copy and locus numbers of 5S rDNA between the three gnetophyte genera (ranging from ~105 000 copies at >12 sites in *G. montanum* to only ~3800 copies at two sites in *W. mirabilis*; Table 2) one would predict the greatest sequence heterogeneity in *G. montanum*. Indeed, this is what was found, with SNPs being at least 10-fold more abundant in *G. montanum* than *W. mirabilis* (Supplementary Data Figs S8A and Table S3). A similar situation was observed for the 35S rDNA, which also differed considerably in copy and locus number between *G. montanum* and *W. mirabilis*. In contrast, *E. altissima* had highly homogeneous 35S and 5S rRNA genes (Fig. 4) despite relatively abundant copies (~6000) and loci (14–16 sites). Perhaps the 35S and 5S rDNA homogeneity in *Ephedra* is related to the L-type arrangement of its rRNA genes, where concerted evolution homogenizes both genes equally and efficiently.

### Frequent amplification of pseudogenes in 5S rDNA units

The occurrence of rDNA pseudogenes is relatively uncommon across eukaryotes, probably because genetic recombination processes act to remove non-functional copies from the tandem arrays (Eickbush and Eickbush, 2007). Therefore it is unusual that two (*G. montanum*, *E. altissima*) out of the three species examined here probably had pseudogenes linked with functional 5S rDNA copies. The large number of highly mutated pseudogenes in *G. montanum* 5S rDNA is reminiscent of the situation in conifers, whose genomes are generally considered to be rich in pseudogenes related to protein-coding genes (Garcia-Gil, 2008; Nystedt *et al.*, 2013). Nevertheless, these data contrast with the generally low pseudogene content identified in the *G. montanum* genome (Wan *et al.*, 2018). However, in that study pseudogenes were scored mostly among protein-coding sequences dispersed around the genomes. Perhaps pseudogenes are better maintained in tandemly repeated families than when dispersed. The retention of 5S rDNA pseudogenes could be related to a relatively recent and massive amplification of an rDNA unit bearing a pseudogenized 5S copy in its NTS spacer. Although Southern blot hybridization profiles support the presence of alternating functional and non-functional copies in the *G. montanum* genome with a tentative structure gm5Sc–gmNTS1–gm5Sp–gmNTS2, the long-range organization of 5S rDNA remains uncertain. This is because assemblies of tandem repeats from short reads may yield artificial contigs whose structures may not necessarily exist in the genome. Thus we also cannot exclude the possibility that functional and pseudogenized 5S rDNA copies might also be separated in different loci. Analysis of long PacBio reads and/or PCR anchored to genes/pseudogenes will be needed to address these questions.

In contrast to *G. montanum*, the intragenomic homogeneity of the pseudogenes in *E. altissima* was comparable to that of the functional genes, suggesting that both pseudogenes and functional genes (within the linked 5S–35S rDNA units) homogenize across the chromosome array, perhaps together, and appear to be evolving in concert. In the angiosperm genus *Artemisia* (Asteraceae), only a few 5S–35S L-type rDNA units have 5S rRNA pseudogenes linked to functional copies, perhaps because selection has acted against pseudogenes (Garcia *et al.*, 2009). In contrast, in *E. altissima* (this study) and *E. major* (Garcia and Kovařík, 2013) all (or nearly all) 5S–35S rDNA units carry pseudogenes (in addition to the functional copies), possibly due to a reduced selection pressure against 5S rRNA pseudogenes in these species.

### rDNA evolution in gnetophytes and its relationship to genome size

The genome sizes in gnetophytes have a range of ~20-fold (http://data.kew.org/cvalues/), with species in *Gnetum* having the smallest genomes so far reported for any gymnosperm (average 1C = 3.5 Gb, ranging from 2.2 to 4.2 Gb/1C) (Bennett and Leitch, 2012; Wan *et al.*, 2018), whilst species in *Ephedra* have the largest genomes [average 1C >15.3 Gb, ranging from 7.9 to 37.5 Gb/1C (Bennett and Leitch, 2012; S. Ickert-Bond *et al*., University of Alaska, USA, unpubl. res.)]. Previously a general correlation across eukaryotes between genome size and rDNA copy number (Prokopowich *et al.*, 2003) and rDNA locus number (Garcia *et al.*, 2017) was found, although there are exceptions (e.g. Hidalgo *et al.*, 2017). Indeed, this trend is also not apparent in the three gnetophytes studied here. *Gnetum montanum* (1C = 4.2 Gb) has a relatively small genome but with >100 000 5S rDNA units, whilst *E. altissima*, with a much larger genome (1C = 18.5 Gb), has ~6000 copies of the 5S–35S linked array (Table 2). *Welwitschia mirabilis* (1C = 7.0 Gb) has only moderate numbers of rDNA copies (5S and 35S) and a single locus of each.

### Conclusions

Gnetophytes with unclear and controversial phylogenetic relationships to other seed plants, distinct morphologies and diverse growth habitats for gymnosperms, possess also astonishingly variable rDNA characteristics. These characteristics include different genomic organizations, unit structure, copy numbers, pseudogene content and intragenomic homogeneity (Fig. 6). These observations may suggest considerable evolutionary distances between its three families, i.e. Gnetaceae, Ephedraceae and Welwitschiaceae, perhaps contributing to the diverse features of their rDNAs.

## SUPPLEMENTARY DATA

Supplementary data are available online at https://academic.oup.com/aob and consist of the following. Figure S1: sequence alignment of 35S rDNA and sequence alignment of 5S rDNA genic sequences. Figure S2: length variation in the different regions of rDNA coding regions and 5S rDNA regions. Figure S3: dot-plot displays of self-to-self alignments of rDNA units. Figure S4: conserved ITS1 structures in *Ephedra*. Figure S5: phylogenetic relationships between ITS1 sequences of *Ephedra*. Figure S6: schematic illustration of the alignment of three non-transcribed spacers (NTSs) in the *W. mirabilis* 5S rDNA unit. Figure S7: 5S rDNA contigs and secondary (2-D) structures of rRNA molecules in *E. altissima*, *E. major*, *G. gnemon*, *G. montanum* and *W. mirabilis*. Figure S8: frequency of SNPs in rDNA sequences of three gnetophytes. Figure S9: dot-plot comparisons of the ITS1 sequences in *E. altissima* and *Pinus cembra*. Figure S10: quantification of rDNA copies by slot blot hybridization. Table S1: calculation of rDNA copy number from the HTS data. Table S2: list of *Ephedra* accessions used in phylogenetic and structural analyses. Table S3: mutation analysis of rDNA in *E. altissima*, *G. montanum* and *W. mirabilis*.

mcy172_Supplementary_Figure_S1

mcy172_Supplementary_Figure_S2

mcy172_Supplementary_Figure_S3

mcy172_Supplementary_Figure_S4

mcy172_Supplementary_Figure_S5

mcy172_Supplementary_Figure_S6

mcy172_Supplementary_Figure_S7

mcy172_Supplementary_Figure_S8

mcy172_Supplementary_Figure_S9

mcy172_Supplementary_Figure_S10

mcy172_Supplementary_Table_S1-S3
