# Supplementary material for: Remarkable variation of ribosomal DNA organization and copy number in gnetophytes, a distinct lineage of gymnosperms
Source: Ann Bot. 2018 Sep 27;123(5):767–81. doi: 10.1093/aob/mcy172 (PMC6526317; doi:10.1093/aob/mcy172)
Supplement: mcy172_Supplementary_Figure_S1 [file mcy172_supplementary_figure_s1.pptx]

## Slide 1
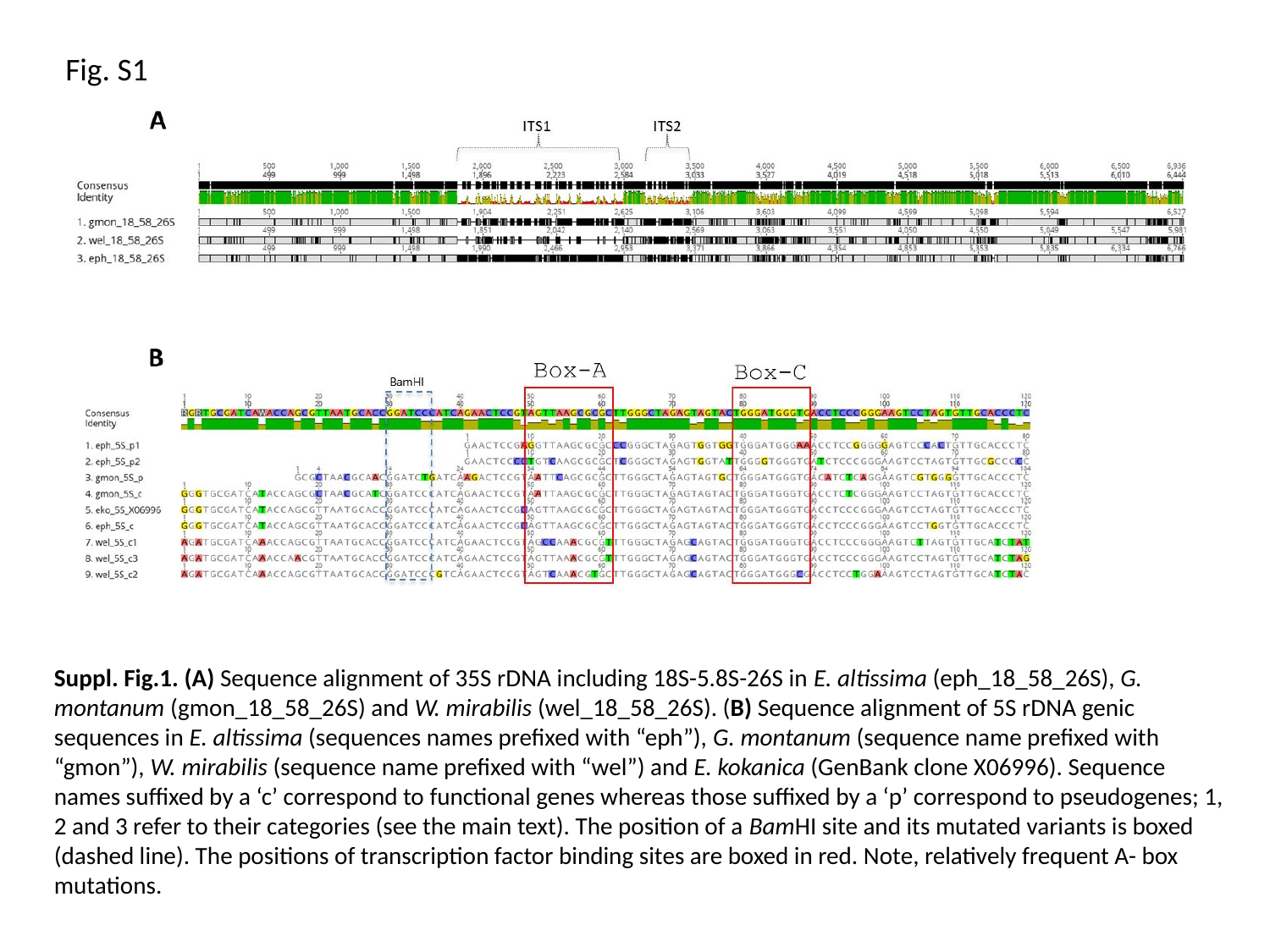

Fig. S1
Suppl. Fig.1. (A) Sequence alignment of 35S rDNA including 18S-5.8S-26S in E. altissima (eph_18_58_26S), G. montanum (gmon_18_58_26S) and W. mirabilis (wel_18_58_26S). (B) Sequence alignment of 5S rDNA genic sequences in E. altissima (sequences names prefixed with “eph”), G. montanum (sequence name prefixed with “gmon”), W. mirabilis (sequence name prefixed with “wel”) and E. kokanica (GenBank clone X06996). Sequence names suffixed by a ‘c’ correspond to functional genes whereas those suffixed by a ‘p’ correspond to pseudogenes; 1, 2 and 3 refer to their categories (see the main text). The position of a BamHI site and its mutated variants is boxed (dashed line). The positions of transcription factor binding sites are boxed in red. Note, relatively frequent A- box mutations.
