# Supplementary material for: Remarkable variation of ribosomal DNA organization and copy number in gnetophytes, a distinct lineage of gymnosperms
Source: Ann Bot. 2018 Sep 27;123(5):767–81. doi: 10.1093/aob/mcy172 (PMC6526317; doi:10.1093/aob/mcy172)
Supplement: mcy172_Supplementary_Figure_S2 [file mcy172_supplementary_figure_s2.pptx]

## Slide 1
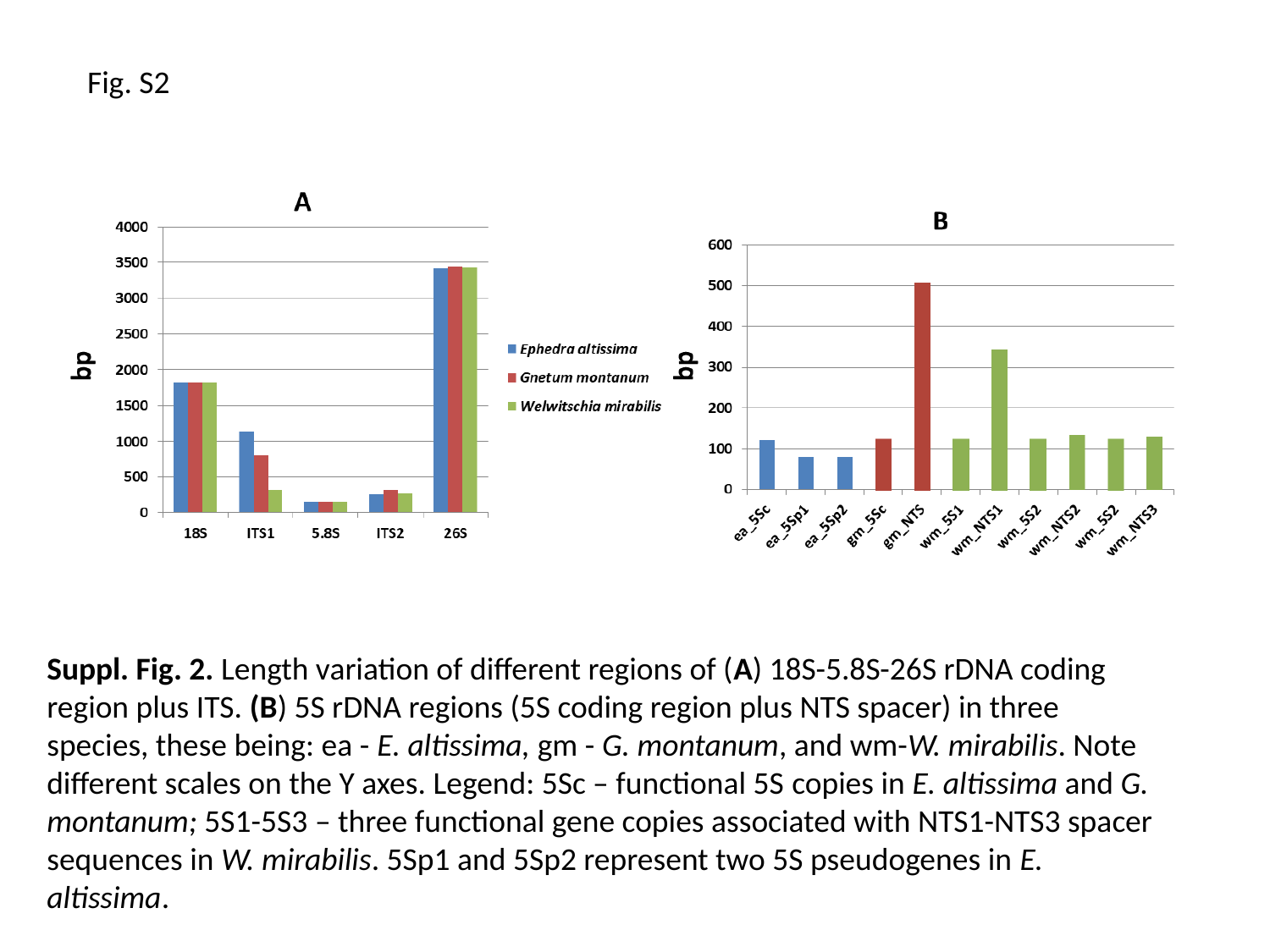

Fig. S2
Suppl. Fig. 2. Length variation of different regions of (A) 18S-5.8S-26S rDNA coding region plus ITS. (B) 5S rDNA regions (5S coding region plus NTS spacer) in three species, these being: ea - E. altissima, gm - G. montanum, and wm-W. mirabilis. Note different scales on the Y axes. Legend: 5Sc – functional 5S copies in E. altissima and G. montanum; 5S1-5S3 – three functional gene copies associated with NTS1-NTS3 spacer sequences in W. mirabilis. 5Sp1 and 5Sp2 represent two 5S pseudogenes in E. altissima.
