# Supplementary material for: Remarkable variation of ribosomal DNA organization and copy number in gnetophytes, a distinct lineage of gymnosperms
Source: Ann Bot. 2018 Sep 27;123(5):767–81. doi: 10.1093/aob/mcy172 (PMC6526317; doi:10.1093/aob/mcy172)
Supplement: mcy172_Supplementary_Figure_S3 [file mcy172_supplementary_figure_s3.pptx]

## Slide 1
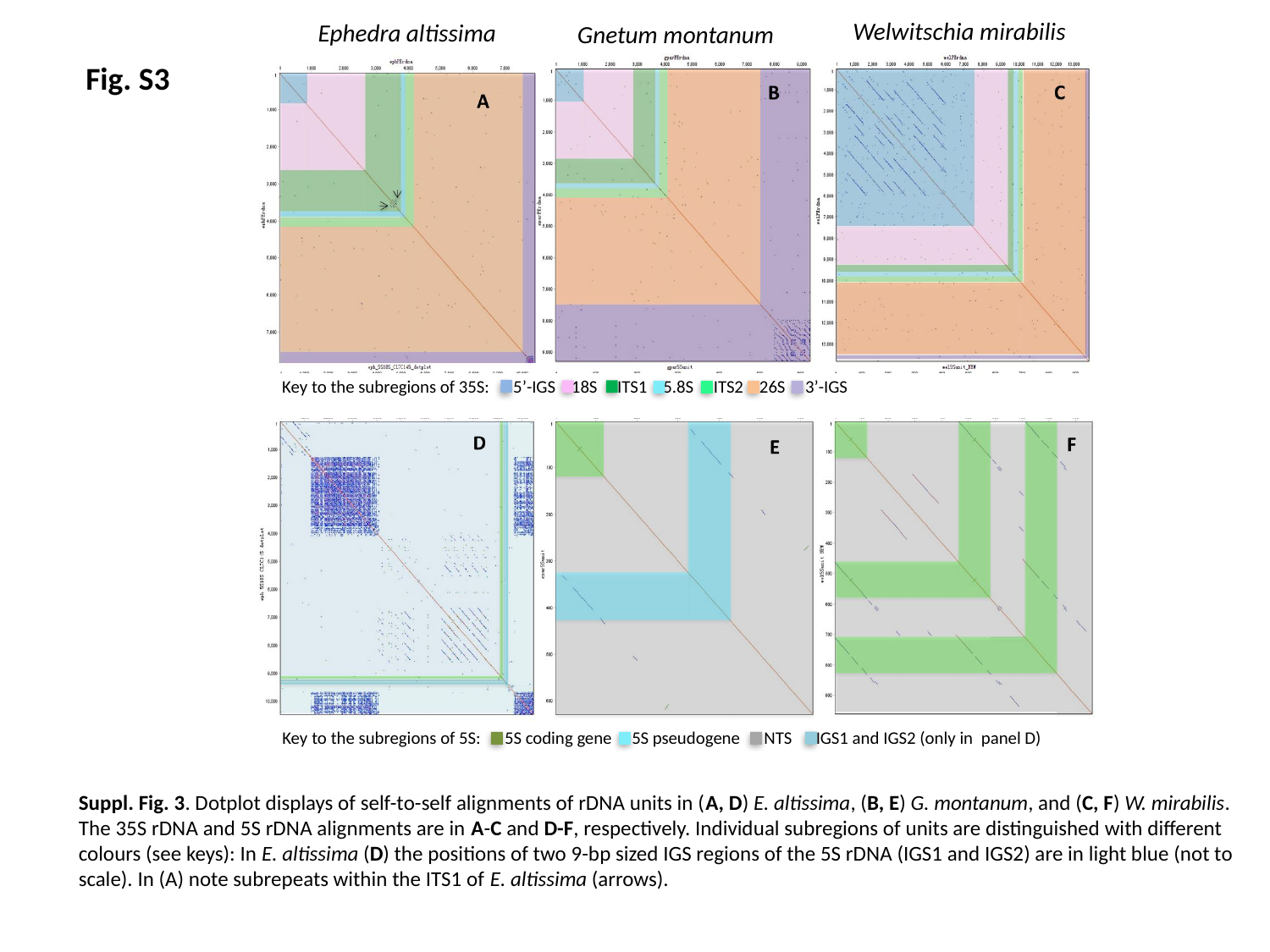

Welwitschia mirabilis
Ephedra altissima
Gnetum montanum
Fig. S3
Key to the subregions of 35S: 5’-IGS 18S ITS1 5.8S ITS2 26S 3’-IGS
Key to the subregions of 5S: 5S coding gene 5S pseudogene NTS IGS1 and IGS2 (only in panel D)
Suppl. Fig. 3. Dotplot displays of self-to-self alignments of rDNA units in (A, D) E. altissima, (B, E) G. montanum, and (C, F) W. mirabilis. The 35S rDNA and 5S rDNA alignments are in A-C and D-F, respectively. Individual subregions of units are distinguished with different colours (see keys): In E. altissima (D) the positions of two 9-bp sized IGS regions of the 5S rDNA (IGS1 and IGS2) are in light blue (not to scale). In (A) note subrepeats within the ITS1 of E. altissima (arrows).
