# Supplementary figures and images for: Remarkable variation of ribosomal DNA organization and copy number in gnetophytes, a distinct lineage of gymnosperms
Source: Ann Bot. 2018 Sep 27;123(5):767–81. doi: 10.1093/aob/mcy172 (PMC6526317; doi:10.1093/aob/mcy172)

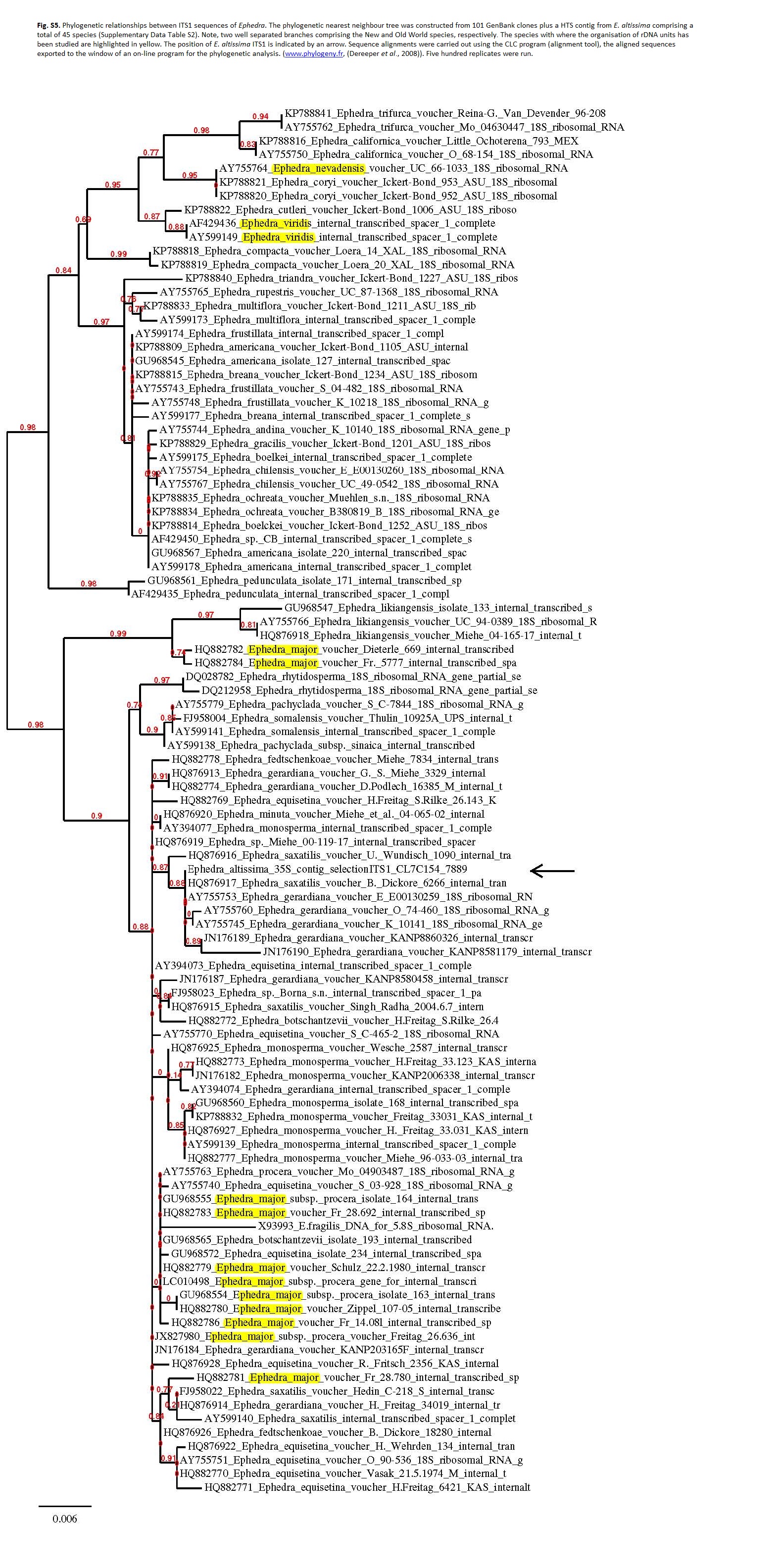

Supplement: mcy172_Supplementary_Figure_S5 [file mcy172_supplementary_figure_s5.jpeg]
