# Supplementary material for: Remarkable variation of ribosomal DNA organization and copy number in gnetophytes, a distinct lineage of gymnosperms
Source: Ann Bot. 2018 Sep 27;123(5):767–81. doi: 10.1093/aob/mcy172 (PMC6526317; doi:10.1093/aob/mcy172)
Supplement: mcy172_Supplementary_Figure_S6 [file mcy172_supplementary_figure_s6.pptx]

## Slide 1
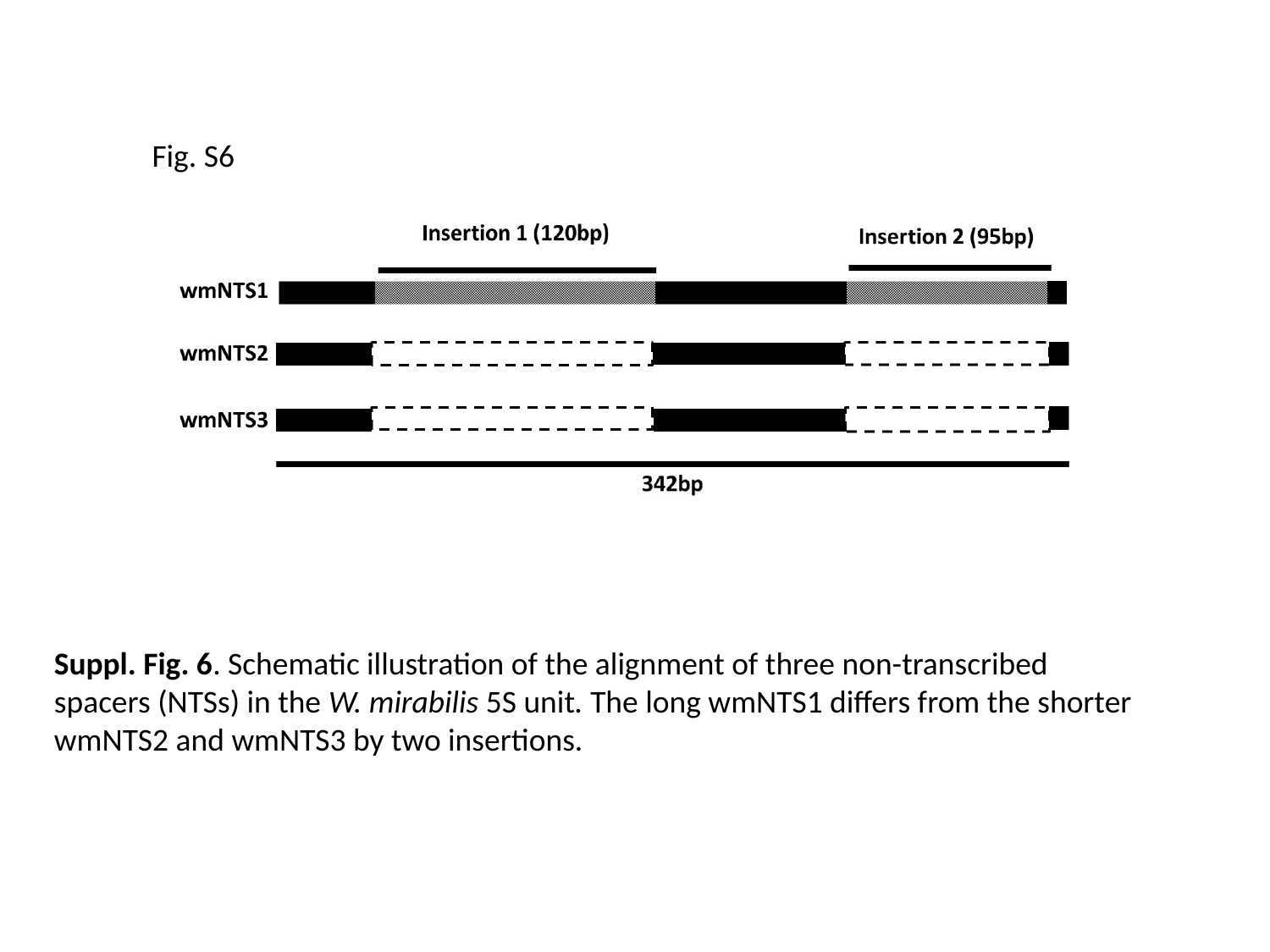

Fig. S6
Suppl. Fig. 6. Schematic illustration of the alignment of three non-transcribed spacers (NTSs) in the W. mirabilis 5S unit. The long wmNTS1 differs from the shorter wmNTS2 and wmNTS3 by two insertions.
