# Supplementary material for: Remarkable variation of ribosomal DNA organization and copy number in gnetophytes, a distinct lineage of gymnosperms
Source: Ann Bot. 2018 Sep 27;123(5):767–81. doi: 10.1093/aob/mcy172 (PMC6526317; doi:10.1093/aob/mcy172)
Supplement: mcy172_Supplementary_Figure_S7 [file mcy172_supplementary_figure_s7.pptx]

## Slide 1
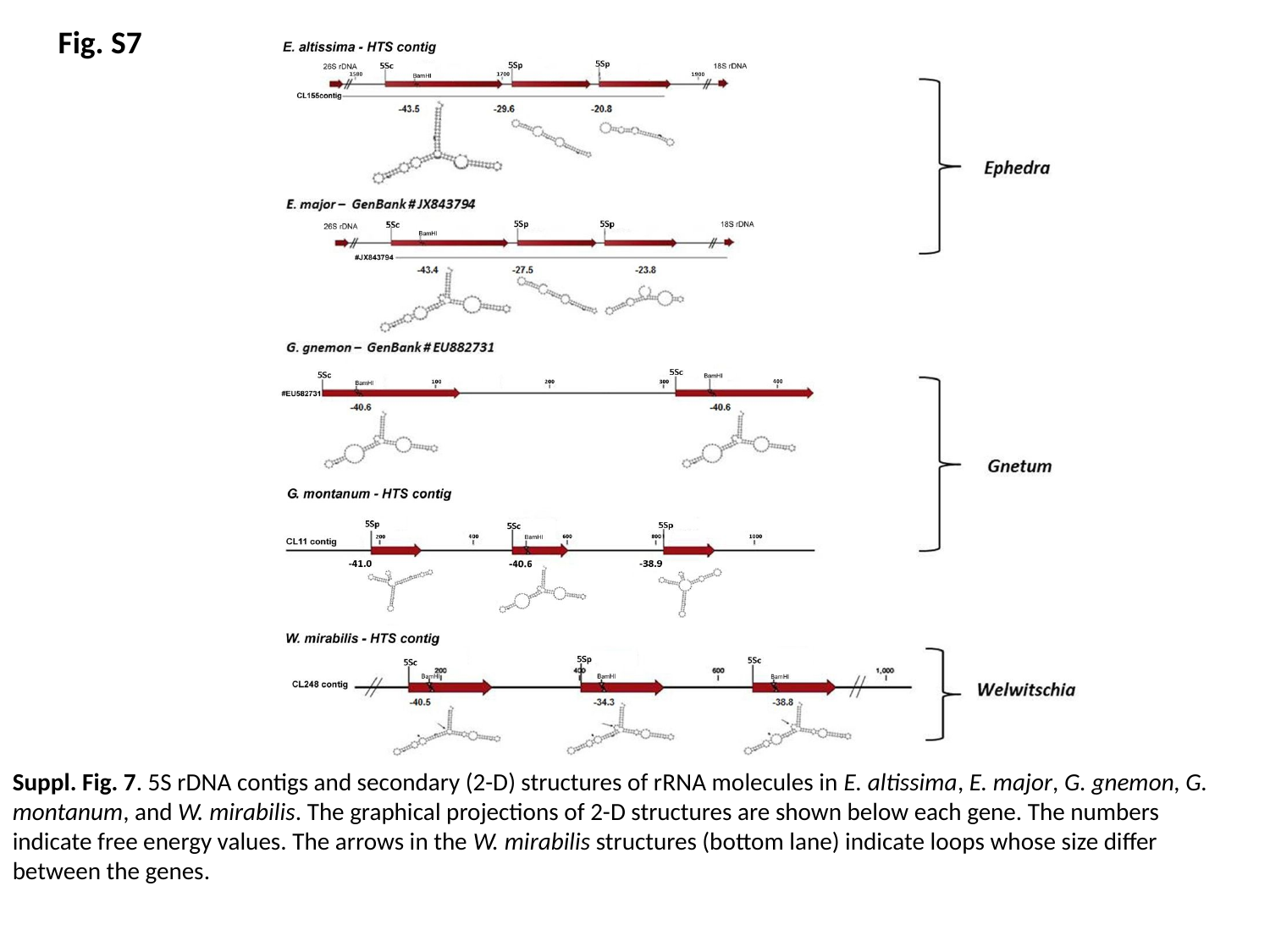

Fig. S7
Suppl. Fig. 7. 5S rDNA contigs and secondary (2-D) structures of rRNA molecules in E. altissima, E. major, G. gnemon, G. montanum, and W. mirabilis. The graphical projections of 2-D structures are shown below each gene. The numbers indicate free energy values. The arrows in the W. mirabilis structures (bottom lane) indicate loops whose size differ between the genes.
