# Supplementary material for: Remarkable variation of ribosomal DNA organization and copy number in gnetophytes, a distinct lineage of gymnosperms
Source: Ann Bot. 2018 Sep 27;123(5):767–81. doi: 10.1093/aob/mcy172 (PMC6526317; doi:10.1093/aob/mcy172)
Supplement: mcy172_Supplementary_Figure_S8 [file mcy172_supplementary_figure_s8.pptx]

## Slide 1
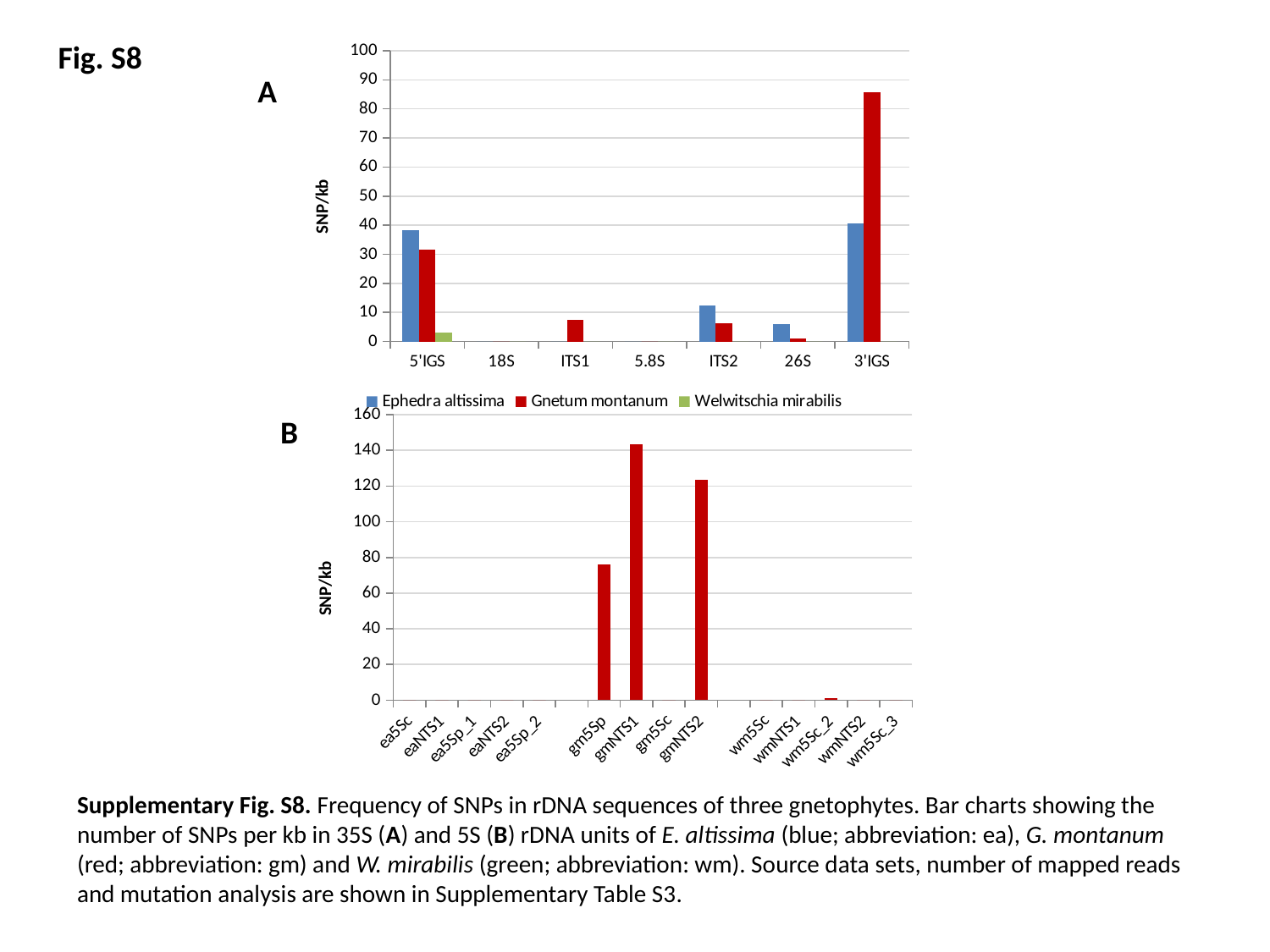

Fig. S8
### Chart
| Category | Ephedra altissima | Gnetum montanum | Welwitschia mirabilis |
|---|---|---|---|
| 5'IGS | 38.186157517899765 | 31.578947368421044 | 3.1270358306188926 |
| 18S | 0.0 | 0.0 | 0.0 |
| ITS1 | 0.0 | 7.537688442211056 | 0.0 |
| 5.8S | 0.0 | 0.0 | 0.0 |
| ITS2 | 12.345679012345686 | 6.349206349206349 | 0.0 |
| 26S | 5.8582308142940835 | 1.1624527753560023 | 0.0 |
| 3'IGS | 40.677966101694885 | 85.74807806031934 | 0.0 |A
### Chart
| Category | |
|---|---|
| ea5Sc | 0.0 |
| eaNTS1 | 0.0 |
| ea5Sp_1 | 0.0 |
| eaNTS2 | 0.0 |
| ea5Sp_2 | 0.0 |
| | None |
| gm5Sp | 76.19047619047612 |
| gmNTS1 | 143.54066985645937 |
| gm5Sc | 0.0 |
| gmNTS2 | 123.71134020618557 |
| | None |
| wm5Sc | 0.0 |
| wmNTS1 | 0.0 |
| wm5Sc_2 | 1.0 |
| wmNTS2 | 0.0 |
| wm5Sc_3 | 0.0 |B
Supplementary Fig. S8. Frequency of SNPs in rDNA sequences of three gnetophytes. Bar charts showing the number of SNPs per kb in 35S (A) and 5S (B) rDNA units of E. altissima (blue; abbreviation: ea), G. montanum (red; abbreviation: gm) and W. mirabilis (green; abbreviation: wm). Source data sets, number of mapped reads and mutation analysis are shown in Supplementary Table S3.
