# Supplementary material for: Remarkable variation of ribosomal DNA organization and copy number in gnetophytes, a distinct lineage of gymnosperms
Source: Ann Bot. 2018 Sep 27;123(5):767–81. doi: 10.1093/aob/mcy172 (PMC6526317; doi:10.1093/aob/mcy172)
Supplement: mcy172_Supplementary_Figure_S9 [file mcy172_supplementary_figure_s9.pptx]

## Slide 1
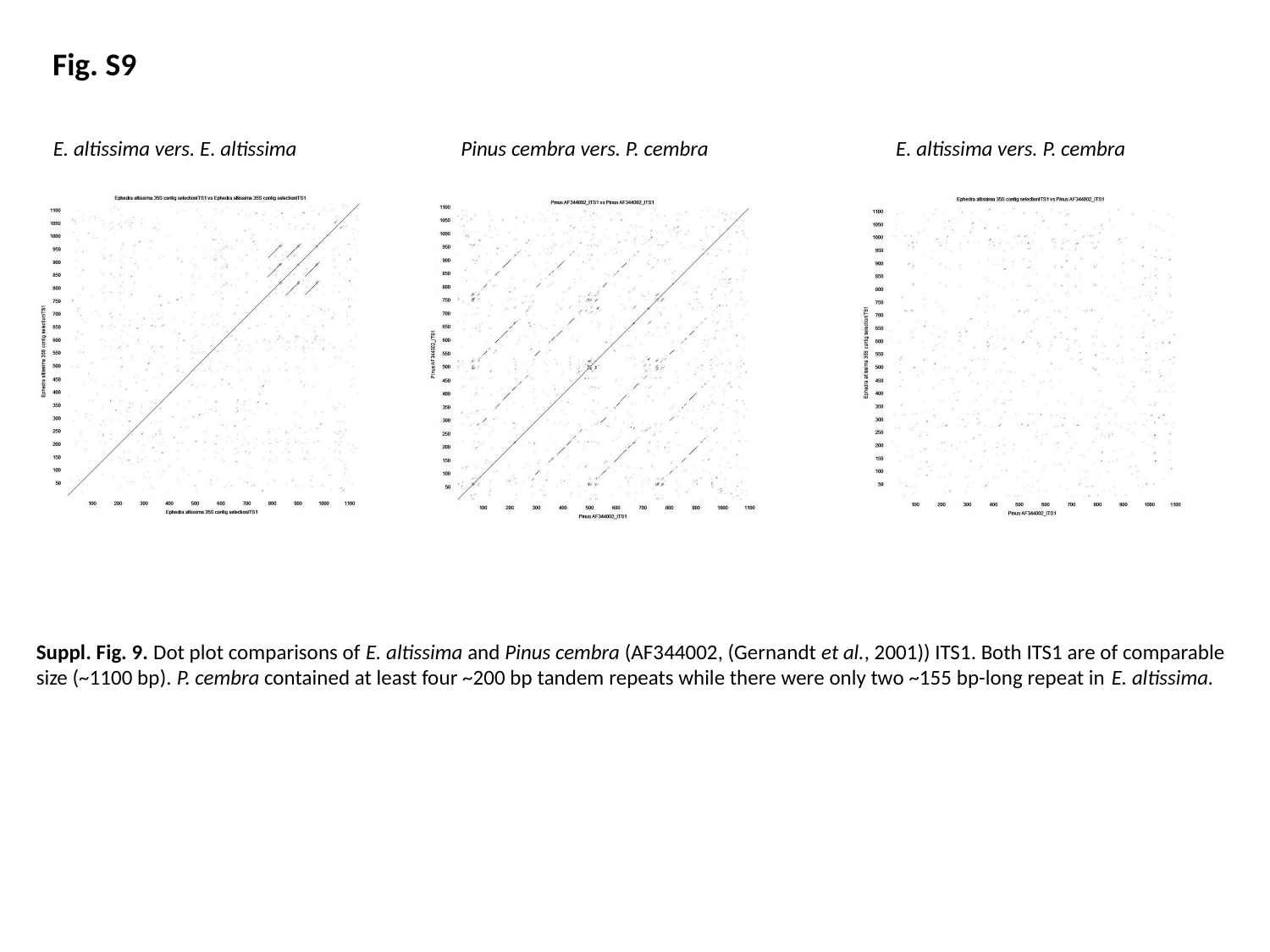

Fig. S9
E. altissima vers. E. altissima
Pinus cembra vers. P. cembra
E. altissima vers. P. cembra
Suppl. Fig. 9. Dot plot comparisons of E. altissima and Pinus cembra (AF344002, (Gernandt et al., 2001)) ITS1. Both ITS1 are of comparable size (~1100 bp). P. cembra contained at least four ~200 bp tandem repeats while there were only two ~155 bp-long repeat in E. altissima.
