# Supplementary material for: Remarkable variation of ribosomal DNA organization and copy number in gnetophytes, a distinct lineage of gymnosperms
Source: Ann Bot. 2018 Sep 27;123(5):767–81. doi: 10.1093/aob/mcy172 (PMC6526317; doi:10.1093/aob/mcy172)
Supplement: mcy172_Supplementary_Figure_S10 [file mcy172_supplementary_figure_s10.pptx]

## Slide 1
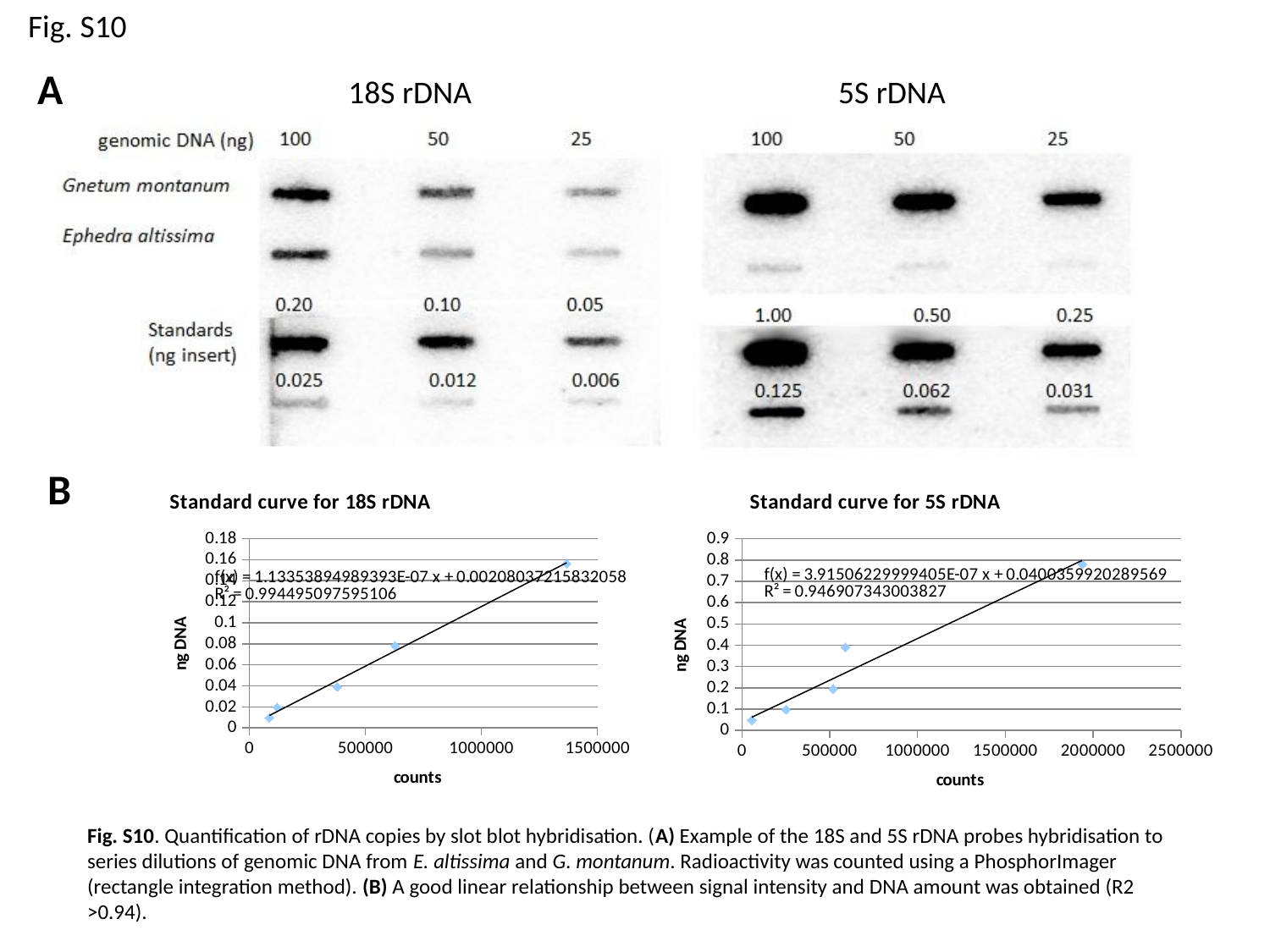

Fig. S10
A
18S rDNA
5S rDNA
B
### Chart: Standard curve for 18S rDNA
| Category | |
|---|---|
### Chart: Standard curve for 5S rDNA
| Category | |
|---|---|Fig. S10. Quantification of rDNA copies by slot blot hybridisation. (A) Example of the 18S and 5S rDNA probes hybridisation to series dilutions of genomic DNA from E. altissima and G. montanum. Radioactivity was counted using a PhosphorImager (rectangle integration method). (B) A good linear relationship between signal intensity and DNA amount was obtained (R2 >0.94).
